# The circular economy and the Green Jobs creation

**DOI:** 10.1007/s11356-021-16562-y

**Published:** 2021-10-03

**Authors:** Adam Sulich, Letycja Sołoducho-Pelc

**Affiliations:** Faculty of Management, Wroclaw University of Economics and Business, Wrocław, Poland

**Keywords:** Green Jobs, Sustainable development, Environmental goods and services sector, Sustainable Development Goals

## Abstract

The circular economy (CE) is a proposal for a new, more sustainable, and durable economy model. As a consequence, this pro-environmental economic model induces visible changes in the labor market which are Green Jobs (GJs). This paper is focused on the creation of Green Jobs in the CE. The GJs are most visible in the environmental goods and services sector (EGSS). This study aims to investigate EGSS among 28 European Union countries in the years 2009–2019. The adopted method was literature research complemented by the statistical analysis of secondary data from Eurostat in the linear regression method. Then, some Sustainable Development Goals (SDGs) and their measure were used as main indicators reflecting changes in the labor market. Results are presented as a model indicating which of the SDGs can support CE and enhance a number of the Green Jobs. Presented results contribute to the science because combine factors influencing GJs creation in EGSS, in a CE perspective. This study underlines a lack of uniform methods for measuring and forecasting the effects of Green Jobs creation and indicates future research directions.

## Introduction

The transition from the linear economy to the circular economy (CE) is a process observed both in theory and practice (Ferasso et al. 2020; Gottinger et al. 2020; de Oliveira et al. 2021). The CE can be summarized as a paradigm shift aimed at preventing the depletion of resources (Barreiro-Gen and Lozano 2020) by closing the loops related to energy and materials consumption (Lozano-Lunar et al. 2020; Rincón-Moreno et al. 2020; O’Connor 2021). This concept can be characterized at the micro- (customers and companies), meso- (economic agents that integrate into symbioses), and macro-levels(from national to regional and city-levels) (Prieto-Sandoval et al. 2018).

Striving for sustainable development (SD) is at the basis of creating a contemporary socio-economic policy of the EU and many countries around the world (Ledoux et al. 2005; Tortorella et al. 2020). This idea of economic development is based on the principle of maintaining synergy and a balance between social, economic, and environmental dimensions (Friant et al. 2021). The key role of the SD is crucial in the EU and is visible in multiple documents (Gottinger et al. 2020). Many EU action plans already referred to the SD and emphasized the need to create more sustainable and inclusive growth in Europe (Bartniczak and Raszkowski 2019; Salvioni and Almici 2020). The EU’s documents concern not only the transition to the CE but also Green Jobs creation (Friant et al. 2020). The CE is also a solution for the environmental problems that expanded nowadays. The CE evolved from the linear economy (LE) often called brown economy (BE) which is an economic model based on the extensive combustion of fossil fuels (Briguglio and Brown 2019).

The research gap identified in this paper is the lack of the Green Jobs creation econometric model connections with the CE. Such a model should describe between theoretical assumptions of CE and empirical implications in the emerging GJs using indicators constructed upon the Sustainable Development Goals (SDGs).

This research intends to fulfill indicated gap by investigating the EGSS in EU countries from 2009 to 2019. Therefore, the scope of the paper is focused on the GJs in the context of CE. The GJs are most visible in the environmental goods and services sector (EGSS). What is more, the scientific literature refers to EGSS as the “green sector” (Sinclair-Desgagné 2008). The comprehensive desk research to complement the statistical analysis of secondary data from Eurostat for each member state was performed (Eurostat 2020a).

This manuscript is structured as follows. After the research gap and aim of this paper are presented in the introduction, the themes crucial for this research are discussed in the literature review in the second point. The research method is described in the third section, then the results and their discussion are presented. The paper concludes by presenting limitations of the conducted research that were discussed along with the managerial implication and contribution to the knowledge development, and further research directions are addressed.

## Literature review

### Circular economy

The CE concept identifies new opportunities for the simultaneous achievement of environmental benefits and economic growth (Hopkinson et al. 2018; Durán-Romero et al. 2020; van Dam et al. 2020). The idea of CE was based on the observation of natural ecosystems, which are not linear (only energy flows), but resources (materials) circulate (Korhonen et al. 2018; Ilić et al. 2020). Then, all production processes should be devised in such a way to be more like natural closed cycles (Kirchherr et al. 2017).

Pearce et al. (1989) in their report entitled “Blueprint for a green economy” presented and explained how to introduce the SD idea in social and economic processes. The authors of this report have not explicitly defined the CE, underlining their idea that the economy should support the natural environment protection policy. According to Green and McCann (2011), a CE is defined as the concept of an environmentally friendly economy that opens new opportunities (Iacovidou et al. 2021) for creative and innovative activities. In 2020, Hasanli indicated that the CE is the future for the world (Hassanli et al. 2020) based on technological progress. Therefore, the term “green” denotes activities considered essential and beneficial to the environment (Whitmarsh and O’Neill 2010). These views on CE are significantly influenced by the growing interest in the SD (Sauvé et al. 2016). As a result of this increased attention reflected in many scientific studies (Sanguino et al. 2020) and governments’ documents, different concepts and derivative definitions were founded (Kirchherr et al. 2017).

The CE is defined as an alternative for the LE (Sulich 2018; Sulich and Zema 2018; Robaina et al. 2020) because CE aims to balance economic activities by closing the loops and creating an ecological system (Boulding 1966; Ruiz-Real et al. 2018; Sanguino et al. 2020). The CE characteristics are often presented in contrast to the linear model of the LE (Table 1). The CE is considered to be a more sustainable, inclusive, and pro-environmental model of growth and development (Moraga et al. 2019; Sanguino et al. 2020). The LE as a model of economic development has failed because it leads toward the destruction of biodiversity and resources depletion. As a linear system, the LE is based on the false assumption that planet resources are unlimited (Corrêa and Corrêa 2021). The LE caused three main consequences (Baer et al. 2015; Sulich and Zema 2018):

1. Environmental burden resulting from industrialization;
2. Political and bureaucratic inefficiency of the public sector;
3. Increasing income, cultural, racial, and ethnic differences.

**Table 1:** Comparison between circular economy versus linear economy features

Circular economy	Linear economy
Separates the economic growth from the natural resources use	“Unlimited” economic growth
Renewable energy sources	Non-renewable energy sources
Energy efficiency	Massive consumption of natural resources (energy and raw materials)
Clean production	Greenhouse gas emissions
Biodiversity protection	Biodiversity destruction
Intergenerational and interregional justice	Creates social inequalities at the global scale
Sustainable consumption	Unlimited consumption (overconsumption)
Corporate social responsibility from companies and stakeholders	Lack of corporate social responsibility from companies and stakeholders
Rising social trust	Awareness of social trust

Source: elaborated based on (Sulich and Zema 2018)

**Fig. 1:**
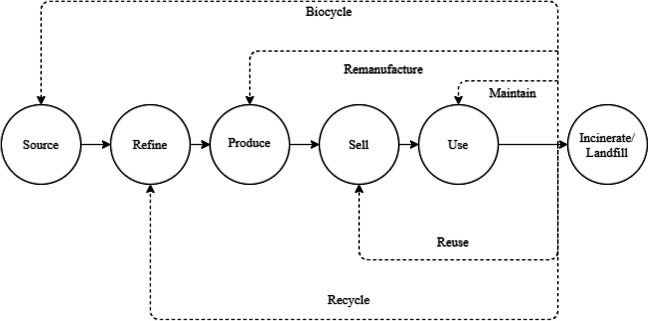
Product life cycle and its linear and circular economy implications. Source: Authors’ elaboration

The CE is a foundation of the “green civilization” concept, which is characterized by the human and natural environment coexistence, by the harmonious social development, and by the technological innovation in the EGSS (Norgaard 1994). As presented in Figure 1, the CE develops not only qualitatively but also quantitatively—the new processes and jobs are created to cover material cycles (Gottwald 2012; Tomić and Schneider 2020).

The CE is a proposal for a more sustainable and durable economic model (Castillo Castillo and Angelis-Dimakis^2019^). What is more, the CE has defined processes that close resources loops (i.e., by recycling) and aims to reduce material losses by incineration and landfill (Hopkinson et al. 2018; O’Connor 2021). The circular economy is a path of economic and social development that relies on investing in the “green recovery.” It means a transition from a LE in favor of creating Green Jobs and the shift toward the CE visible in the environmental goods and services sector.

### Environmental goods and services sector in European Union

The definition of the EGSS, which is used in this paper, was published in 2009 in the “*EGSS handbook*” (Broniewicz and Domańska 2016) in explaining what the environmental goods and services are. In the proposed approach, these goods and services related to environmental protection are distinguished based on the main goal’s criterion. The EGSS aim is to protect the environment or manage resources (Moldvay et al. 2013; Broniewicz and Domańska 2016). “Also critical is the producer’s awareness of environmental requirements and the creation of environmentally friendly products, the use of products in harmony with the environment, and offering them in markets that take environmental conditions into account” (Eurostat 2015; Broniewicz and Domańska 2016). The definition of EGSS is also stated in Regulation (EU) No. 691/2011, when classifying environmental activities based on the objective criterion. These actions area can be divided into environmental protection (EP) and resource nanagement (RM) activities (Steuer et al. 2016; Eurostat 2020b). Environmental goods are related to environmental services as they arise from environmental processes. Then environmental products can be the primary, secondary or auxiliary manufacturer’s production and serve EP or RM. The green terminology also introduces the environmental producers’ concept, which refers to producers involved in pro-environmental solutions (Broniewicz and Domańska 2016).

The regulation EU No. 691/2011 lists the environmental activities and products under the EGSS (Eurostat 2020b). “The EGSS reporting collects, collates, and reports information on market output (including export), value-added of market activities, employment of market activities” (Eurostat 2020b). Among several outputs, there are the “non-market, for own final use, ancillary environment, and gross value added and employment” (UNEP 2008; Sulich et al. 2020). The EGSS considers the economic activities responsible for generating products which base is the environment, such as those produced for EP or RM. To support initiatives related to EP, the EU Commission has precisely defined environmental products and activities (Sugiyono and Dewancker 2020). “Products for EP prevent, reduce, and eliminate pollution or any other degradation of the environment. They include measures undertaken to restore degraded habitats and ecosystems. Examples are electric vehicles, catalysts, and filters to decrease pollutant emissions, wastewater, and waste treatment services, or noise insulation works. Products for RM safeguard the stock of natural resources against depletion. Examples are renewable energy production, energy-efficient, and passive buildings, seawater desalinization, or rainwater recovery” (Eurostat 2020b).

The EGSS is the sector where Green Jobs are created next to the EP activities (Ernst et al. 2019), which are categorized according to the Classification of Environmental Protection Activities (CEPA). Also related to the EGSS is the list Classification of Resource Management Activities (CReMA) associated with Green Jobs (Eurostat 2020b). These classifications distinguish sixteen categories and subcategories (Eurostat 2020b) which are following the Statistical Classification of Economic Activities in the European Community (NACE 2) (European Commision 2008).

The EGSS meets environmental goals, i.e., industry enterprises’ goods and services help to preventing, reducing, and eliminating ecological degradation or protecting and maintaining natural resources (Livesey 2010; Eurostat 2015).

The EGSS is related to the CE because closing linear chains into loops creates direct and indirect (induced) Green Jobs based on technology development. These expand the EGSS into new EP and RM-related processes and creates new jobs (Karaferye and Agaoglu 2017; Xu et al. 2020). The EGSS is a part of strategic importance in building a CE in the EU (Durán-Romero et al. 2020).

Crowley (1999) notes, in the modern world, enterprises’ orientation toward environmental friendliness should be treated as a higher good. The approach to environmental protection changes, which is not treated as an economic burden, but constitutes a development opportunity (D’Adamo and Lupi 2021) and allows for jobs creation (Crowley 1999).

### Green Jobs in circular economy model

Observed climate and technological changes not only generate threads but also open some opportunities associated with the creation of the Green Jobs (GJs). On the other hand, investments in GJs bring relevant economic, environmental, and social benefits. The benefits of GJs vary, as they generate environmental benefits in traditional and new sectors (Esposito et al. 2014). The GJs creation is associated with a new approach to business management, aiming to prevent environmental degradation and reduce unemployment (Sulich et al. 2020; McMahon et al. 2021). Therefore, GJs are essential for protecting the environment and the labor market, combining sustainability principles (Paes et al. 2019; Unay-Gailhard and Bojnec 2019).

The Green Jobs (GJs) definition used in this article is accepted after the UNEP (2008) term. The Eurostat has not defined GJs explicitly but it appears in the context of the EGSS (Livesey 2010). Despite the lack of the GJs definition in EU documents, its main assumptions are reflected in EU initiatives targeting the so-called balance of two major concerns: the environment and economic growth. The potential of Green Jobs is promising as the CE should help protect the environment and ensures decent work (Toan et al. 2016). The International Labour Organization (ILO) has proposed the term of decent work. “Decent work sums up the aspirations of people in their working lives. It involves opportunities for work that is productive and delivers a fair income, security in the workplace and social protection for families, better prospects for personal development and social integration, freedom for people to express their concerns, organize and participate in the decisions that affect their lives, and equality of opportunity and treatment for all women and men” (ILO 2020). Decent work is recognized as part of the Sustainable Development Goals’ achievement in the formulated by the EU document titled “2030 Agenda for the SD” (United Nations 2021). Therefore, each definition of GJs presented in Table 2 has a multidimensional impact (Pettinger 2017).

**Table 2:** Green Jobs definitions

Organization	Definition
Bureau of Labor Statistics (BLS)	Green Jobs are as follows: a) Jobs related to the goods manufacturing/services providing that can benefit the environment or save natural resources. b) Jobs devoted to establishing environmentally friendly production processes and less use of resources from natural sources made by workers. Categories of green goods/services/technologies are available at the BLS Green Jobs definition.
United Nations Environment Program (UNEP)	“Green Jobs are workplaces in agricultural, manufacturing, research and development, administrative, and service activities that contribute substantially to preserving or restoring environmental quality. Specifically, but not exclusively, this includes jobs that help to protect ecosystems and biodiversity; reduce energy, materials, and water consumption through high-efficiency strategies; de-carbonize the economy; and minimize or altogether avoid the generation of all forms of waste and pollution.” (UNEP 2008)
International Trade Union Confederation (ITUC)	In the context of economic sectors, Green Jobs are responsible for reducing the environmental impacts of such economic activities performed by for-profit companies. Green Jobs also provide the support for decent work and improving workforce living conditions and greater considerations of labor rights.
International Labor Office (ILO)	Green Jobs relate to those that reduce environmental impacts and promote sustainability. Green Jobs include those that are related to the reduction of several consumptions of energy/raw materials, those related to the decarbonization of the economy, those that promote ecosystems and biodiversity protection/restoration, and those that reduce waste and pollution generation. The broad focus of the Green Jobs concept encompasses any new position that shows a smaller than average environmental footprint.
Eurostat	No definition of Green Jobs in the context of EGSS. However, consider EGSS employment for measuring procedures.

Sources: (UNEP 2008; Rutkowska-Podołowska et al. 2016; Bureau of Labor Statistics 2020; Eurostat 2020a; UNEP 2020).

According to Table 2 and Harvey et al. (2010), the organizations create GJs where resources, such as health, time, talent, and money, are not wasted. This new trend responds to the particular need for organizations to be pro-ecologically involved and generating less waste and reducing emissions. Observed changes are a challenge for employers, as it is necessary to reconcile the “eco” and green approaches when managing the organization, making profits, and being competitive (Sołoducho-Pelc and Sulich 2020).

**Fig. 2:**
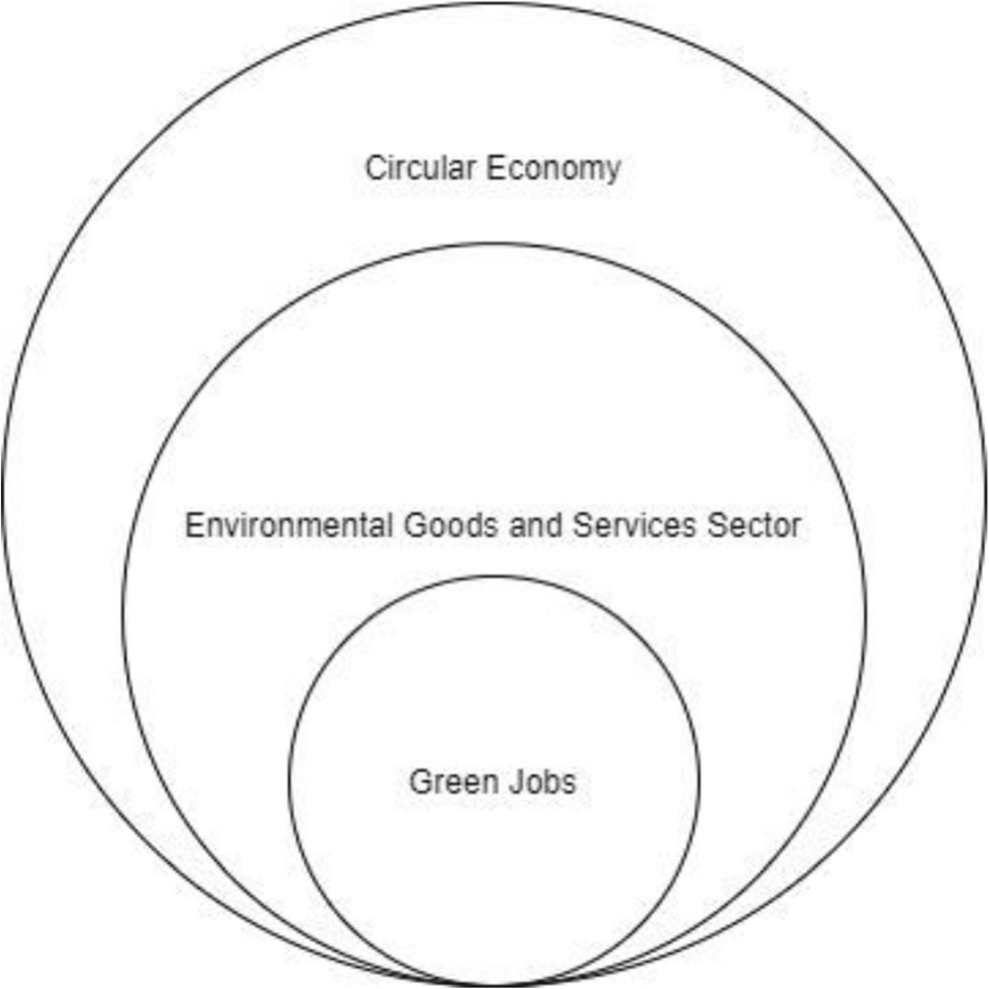
Relations between CE, EGSS, and GJs. Source: Authors’ elaboration

The idea of the CE development and the evolution of SD affects the work and competence of employees. The research carried out by Song and Xie (2019) has already shown that economic development is influenced by the green labor participation rate, the GJs, and green talent (Song and Xie 2019). Therefore, both the needs and expectations of new employee competencies are growing (Burger et al. 2019). Considering the aforementioned contributions, an analytical display of the different definitions relating to GJs as in Figure 2 was presented.

The Green Jobs Initiative (ILO 2021) was created by the international organizations the International Labor Organization (ILO) and the United Nations Environment Program (UNEP) and the International Trade Union Confederation (ITUC). The initiative is covering the impacts caused by climate change on employment and how to mitigate undesirable consequences for future programs. This initiative also supports governments and employers’ efforts in promoting sustainable and environmentally Green Jobs targeting climate change issues. This initiative has a set of goals: “to promote awareness and dialog; to identify and respond to knowledge gaps; to facilitate a “just transition” that reflects the environmental, economic and social pillars of sustainable development; to promote policies and measures to achieve Green Jobs; to catalyze employment and poverty alleviation within climate mitigation and adaptation programs; and to strengthen collaboration between UNEP/ILO/ITUC, within the UN system and with the international business community to establish a common “Green Jobs” definition” (UNEP 2008).

The impact of the GJs on the labor market and employment can be distinguished from a broad conceptual perspective, in at least four ways as the economy is oriented toward greater sustainability and CE. “First, in some cases, additional jobs will be created—as in the manufacturing of pollution-control devices added to existing production equipment. Second, some employees will be substituted—as in shifting from fossil fuels to renewables, or from truck manufacturing to rail car manufacturing, or from landfilling and waste incineration to recycling. Third, certain jobs may be eliminated without direct replacement—as when packaging materials are discouraged or banned and their production is discontinued. Fourth, it would appear that many existing jobs (especially such as plumbers, electricians, metal workers, and construction workers) will simply be transformed and redefined as day-to-day skill sets, work methods, and profiles are greened” (UNEP 2008).

**Fig. 3:**
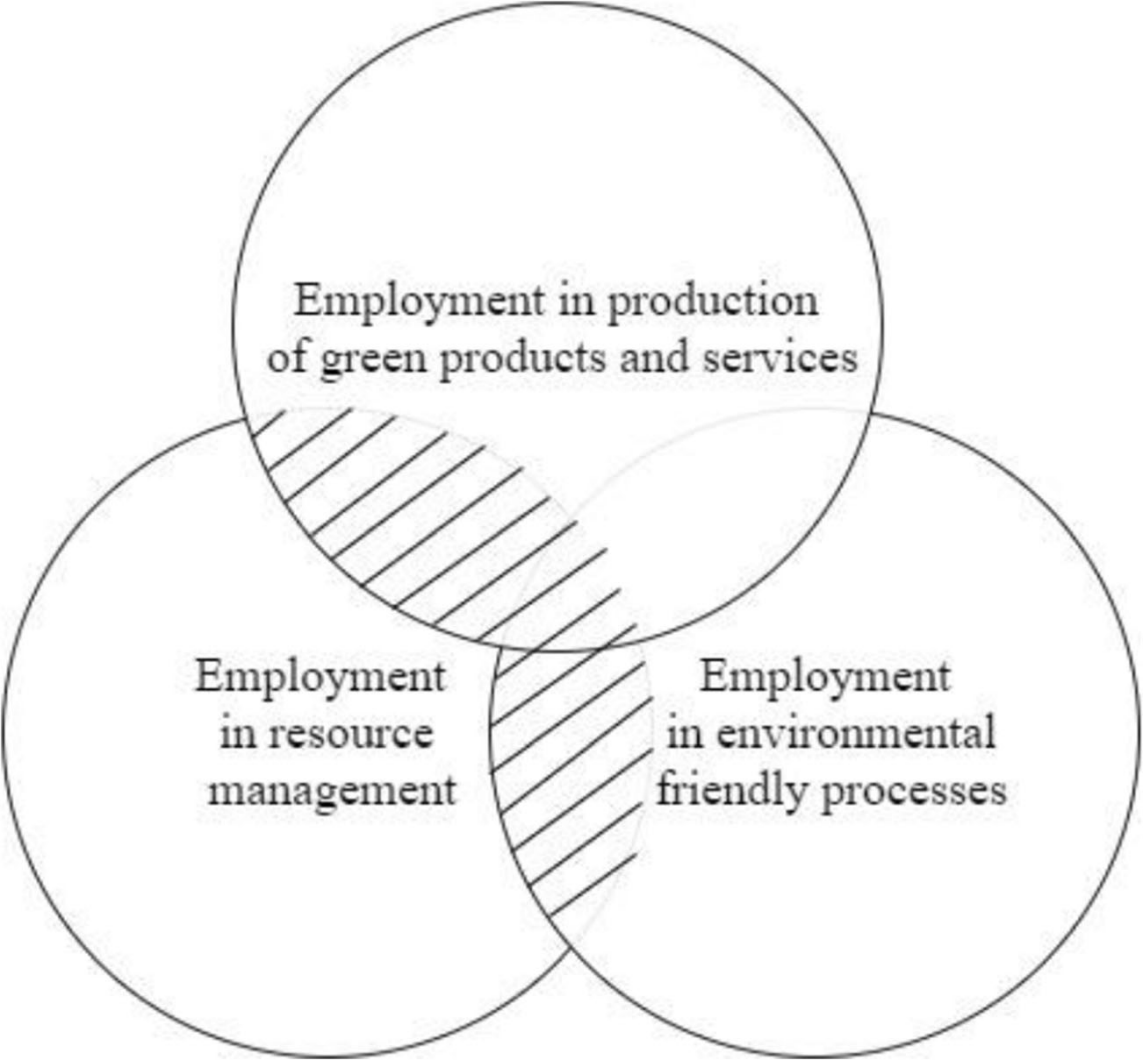
Green Jobs as an area between core elements of revised definitions and EGSS elements Source: (Sulich and Zema 2018).

Figure 3 shows the relations between the three areas of employment: (1) production of green products and services, (2) environmental processes (EP), and (3) resources management (RM). There are GJs in the shaded part of Figure 3 and these are part of the EGSS employment (Livesey 2010). They can be described as pure GJs because these jobs are created in a unique environment created by the sector related directly to the natural environment (Sulich and Zema 2018).

Presented in Table 2 definitions can be operationalized based on two core elements, which are consistent with the output and process approaches (Bureau of Labor Statistics 2020). In the EGSS context, the EP and RM approaches bring focus on the goods and services outputs and indicators suitable for the CE (Piwowar-Sulej 2021). Additionally, the problem of measurability and objectivity in evaluation appears in scientific research and reporting on the GJs worldwide, and in this context, explanatory model combining CE and GJs is required (Gagliardi et al. 2016; Dordmond et al. 2021).

## Research design

The adopted research method is the statistical linear regression method. This method is mostly used in the research of the economic development comparisons (Kasztelan 2016; Raszkowski 2018; Moraga et al. 2019; Sulich et al. 2020) related to the usage of the full set of variables to create an econometric model. In this research data obtained from the Eurostat database for all 28 EU countries in years, 2009–2019 were used, because of their reliability (Eurostat 2020c). What is more, the EGSS data are made available every year, and these data are also part of the sustainable development indicators (more than 100 overlapping variables in a very wide context), gathered in the Eurostat database grouped in 17 sustainable development goals (SDGs) (Schroeder et al. 2019; Rincón-Moreno et al. 2020). Then, among these indications, there is also a distinguished group of the CE which consists of 15 indicators. In the research method, they are considered as the describing independent variables (with symbols from CE_1 to CE_15) to the variable with symbol GJs—Green Jobs (described variable). All variables related to the CE and EGSS employment were identified for further calculations performed with the Statistica® Software. Presented in Table 3, variables are given by Eurostat to measure CE transition among EU countries and were not the result of the authors’ choice. The Green Jobs variable (GJs) which employment is in the EGSS is adopted in this paper. Data on EGSS are widely applicable and are used in the economy, politics, and social activities (Demidova et al. 2021). Both in microeconomics and macroeconomics, these data are used to formulate environmental management goals and to monitor them (Eurostat 2015). In Table 3, there are three variables marked with * symbol, which are not used in calculations due to their characteristics: CE_1 (cei_pc010); CE_7 (cei_wm020); and CE_11 (cei_srm010). These variables are not associated with EU member states, but with the specified resources and are listed by Eurostat to describe CE. Therefore, continuous enumeration of these indicators was kept in accordance with the Eurostat database (Eurostat 2020c).

**Table 3:** Chosen indicators measured by the Eurostat (Eurostat 2020d)

Indicator group	Variable symbol	Indicator characteristic	Eurostat symbol
Production and consumption	CE_1*	EU self-sufficiency for raw materials	(cei_pc010)
	CE_2	Generation of municipal waste per capita	(cei_pc031)
	CE_3	Generation of waste excluding major mineral wastes per GDP unit	(cei_pc032)
	CE_4	Generation of waste excluding major mineral wastes per domestic material consumption	(cei_pc033)
Waste management	CE_5	Recycling rate of municipal waste	(cei_wm011)
	CE_6	The recycling rate of all waste excluding major mineral waste	(cei_wm010)
	CE_7*	The recycling rate of packaging waste by type of packaging	(cei_wm020)
	CE_8	Recycling rate of e-waste	(cei_wm050
	CE_9	Recycling of biowaste	(cei_wm030)
	CE_10	The recovery rate of construction and demolition waste	(cei_wm040)
Secondary raw materials	CE_11*	Contribution of recycled materials to raw materials demand -end-of-liferecycling input rates (EOL-RIR)	(cei_srm010)
	CE_12	Circular material use rate	(cei_srm030)
	CE_13	Trade in recyclable raw materials	(cei_srm020)
Competitiveness and innovation	CE_14	Private investments, jobs, and gross value added related to circular economy sectors	(cei_cie010)
	CE_15	Patents related to recycling and secondary raw materials	(cei_cie020)
Labor market	GJs	Employment in the environmental goods and services sector	(env_ac_egss1)

Source: Authors elaboration based on (Eurostat 2020e, 2020f)

*Data were unavailable or not in the geographical breakdown suitable for further research

The presented division of the variables is in order with the method adopted by the Eurostat (Eurostat 2020c). The performed multiple regression aim was to create the econometric model to cover described in the introduction section research gap and to propose a reliable model based on the variables proposed by Eurostat.

## Data analysis and discussion

This paper has researched the secondary data related to the CE indicators gathered for each EU member state in years 2009–2019 and published in the Eurostat database in a dedicated section entitled “Circular economy indicators” (Eurostat 2020f). The two sets of variables in a total number of 13, which were based on the Eurostat database were defined. The first set of 12 variables (symbols as in Table 3) are describing and independent variables when the one variable with symbol GJs is dependent and described variable.

The correlation (Table 4) and basic statistics (average and standard deviation values) for unstandardized data, aiming to check interdependencies between variables were examined. Moreover, it is intended to find out the directions of the relations of the aforementioned variables. The main research assumption is the correlation examination of variables possesses a sense only a cause-and-effect relation is present. This research used calculations from the Statistica® software made available by StatSoft Poland programming environment. The dependent variable GJs is significantly correlated with variables: CE_5, CE_9, CE_12, CE_13, CE_14, and CE_15, as presented in Table 4.

**Table 4:** Correlations matrix between variables (circular economy indicators, CE) and Green Jobs (variable) for all EU-28 countries

Variable	Marked bold correlations are important *p* < 0,05000; *N*=28 (lack of data were deleted by pairs)														
	Average	Std. dev.	CE_2	CE_3	CE_4	CE_5	CE_6	CE_8	CE_9	CE_10	CE_12	CE_13	CE_14	CE_15	GJs
CE_2	482.0	126,0	1,000000												
CE_3	110,2	137,8	−**0,375505**	1,000000											
CE_4	12,3	6.,9	−0,122393	**0,559462**	1,000000										
CE_5	33,0	15,0	**0,431254**	−0,212862	0,277876	1,000000									
CE_6	46,1	20,3	0,231103	−0,354243	0,171651	**0,646556**	1,000000								
CE_8	35,4	14,4	−0,096983	0,208936	−0,032149	0,131294	0,010760	1,000000							
CE_9	56,6	50,0	**0,568365**	−0,334167	0,178549	**0,848741**	**0,611619**	0,024558	1,000000						
CE_10	80,4	23,3	0,174276	−0,024292	−0,001060	0,268182	0,342535	−0,055036	0,247473	1,000000					
CE_12	8,6	6,1	0,117380	−0,007353	**0,699685**	**0,595384**	**0,593859**	−0,141969	**0,582541**	0,213106	1,000000				
CE_13	317705,8	479356,6	0,177678	−0,202030	0,255277	**0,461017**	0,214565	−0,079020	**0,408805**	−0,039978	**0,388470**	1,000000			
CE_14	4721,8	7965,0	0,182528	−0,198114	0,259479	**0,499358**	0,280542	−0,148438	**0,380676**	0,156818	**0,506986**	**0,606161**	1,000000		
CE_15	11,5	19,0	0,121241	−0,161956	0,119587	**0,487828**	0,278448	−0,133799	0,327494	0,163277	**0,410916**	**0,675437**	**0,842345**	1,000000	
GJs	122821,8	148829,8	0,085337	−0,180231	0,189012	**0,442660**	0,299066	−0,203304	**0,394094**	0,209443	**0,476181**	**0,627089**	**0,935198**	**0,863433**	1,000000

Source: Authors’ elaboration.

The regression aimed to identify the primary model based on the reduced number of variables as presented in Table 5.

**Table 5:** Dependent variable GJs regression summary and model characteristics

***N*=28**	b*	Std. Err.b*	***b***	**Std. err.*b***	***t*(21)**	***p***
Intercept			137303,8	46242,92	2,96919	0,007319
CE_14	**0,721735**	**0,101815**	**13,5**	**1,90**	**7,08871**	**0,000001**
CE_15	**0,303899**	**0,102564**	**2386,0**	**805,26**	**2,96302**	**0,000001**
CE_2	−0,169005	0,065845	−199,6	77,76	−2,56671	0,017977
CE_9	**0,388383**	**0,113577**	**1156,7**	**338,27**	**3,41956**	**0,000001**
CE_5	−0,341973	0,112701	−3387,3	1116,32	−3,03434	0,006306
CE_10	0,071692	0,055855	457,5	356,41	1,28354	0,213286

Note: Model features: *R*= 0,96934832; *R*^2^= 0,93963617; Corrected *R*^2^= 0,92238937; *F*(6,21)=54,482; *p*<0.000001; Error std. estimation = 0.41462; symbols meaning: b* standard coefficients, *b* directional coefficient of equation

Source: Authors’ calculations

In Table 5 only significant (*p*<0.000001) variables for the model were marked with bold text, and the others are insignificant: CE_2; CE_5; CE_10, and this can mean that these variables are collinear (CE_5) with the other independent variables or their correlations are weak with the dependent variable (as explained in Table 4).

As a result, the simplified equation was obtained, representing the relations between GJs and its describing variables CE_14, CE_15, and C_9. The linear model formula (linear polynomial) is presented as Eq. (1):

1 $$ \mathbf{GJs}=\mathbf{CE}\_\mathbf{14}\cdotp 13,5+\mathbf{CE}\_\mathbf{15}\cdotp 2386,0+\mathbf{CE}\_\mathbf{9}\cdotp 1156,7\pm 0.4146 $$

**Fig. 4:**
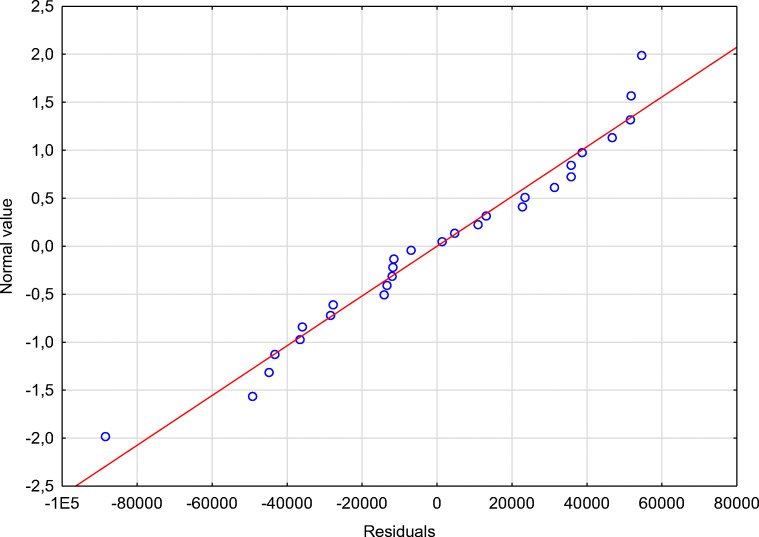
The residuals normality distribution chart. Source: Authors’ calculations

In Eq. 1, the variables’ meaning is the same as in Table 3. The linearity of the presented model was checked in test *F*(6,21) = 54,482. We have verified the model, which consists of checking the model assumptions:

1. The significance of linear regression (note under Table 5) is *p*<0.000001;
2. The importance of partial regression coefficients;
3. No collinearity (redundancy) between independent variables;
4. Homoscedasticity assumption, which means that the variance of the random component (*ε*_*i*_) is the same for all observations;
5. No residual autocorrelation;
6. Normal residual distribution (Figure 4); and
7. The random term *ε*_*i*_ has the expected value equal to 0.

The multiple regression coefficient *R* = 0,96934832 is a measure of the interdependencies between independent variables (12 qualified to research) and dependent variable (Green Jobs).

Then, the obtained calculations allowed the following results (Sołoducho-Pelc and Sulich 2020):

1. Linear regression’s significance was *F* = 54,48 with *p*<0.000001. This result proved that Eq. 1 is significant. The coefficient of multiple correlations was *R* = 0.96, supporting the linear dependencies between variables (Eq. 1);
2. The *p*<0.000001 value evidence the significance of partial regression coefficients;
3. No collinearity between independent variables was verified, with high tolerance values for both variables (close to 1);
4. Model linearity is supported by the fulfilled homoscedasticity;
5. No residual autocorrelation was identified (Table 8);
6. Normal residual distribution was identified (Figure 4); and
7. The random term *ε*_*i*_ reported an expected value (*ε*_*i*_ = 0) thanks to the average value of the Cook’s distance (= 0) (Table 7).

In Table 6, the average values and standard deviations for each variable were calculated. For all variables, all 28 cases (which refer to 28 EU countries) were important to calculate the presented multiple regression model (Table 5). Variables symbols used in Table 6 are the same as in Table 3.

**Table 6:** Variables and their average and standard deviations values

Variable	Average	Std. dev.	No. important
CE_2	482,0	126,0	28
CE_3	110,2	137,8	28
CE_4	12,3	6,9	28
CE_5	33,0	15,0	28
CE_6	46,1	20,3	28
CE_8	35,4	14,4	28
CE_9	56,6	50,0	28
CE_10	80,4	23,3	28
CE_12	8,6	6,1	28
CE_13	317705,8	479356,6	28
CE_14	4721,8	7965,0	28
CE_15	11,5	19,0	28
GJs	122821,8	148829,8	28

Source: Authors’ calculations

The next step of the calculated model verification was to check the residuals normality distribution presented in Figure 4. The plot of the normal distribution of residuals shows that all residuals are arranged along a straight line. The outliers may be the cause of this, and it is assumed that the resulting distribution of residuals is normal.

The statistics shown in Table 7 calculated in regression for variable GJs are used to identify outliers: standardized residuals, residual values removed, Mahalanobis distances, Cook distances. If the observed values among one of these statistics are in the same order, this informs about the lack of outliers. If the observation of calculation results (Table 7) proved differences in these values, then probably, the given case (1–28) and this order is related to the alphabetical order of EU member states as listed in Eurostat tables (Eurostat 2020c) has a significant influence on the regression bias.

**Table 7:** Expected values and residuals in regression for variable GJs (Green Jobs)

	Obs. value	Predict value	Residual	Std. Predict	Std. residual	Std. err predict value	Mahaln dist.	Deleted residuals	Cook dist
1	33876,000000	45305,078125	−11429,078125	−0,537311	−0,275652	27671,212891	11,061666	−20607,994141	0,015719
2	38150,000000	6771,692871	31378,306641	−0,804407	0,756797	14358,762695	2,273865	35654,390625	0,012670
3	113816,000000	77998,562500	35817,437500	−0,310695	0,863862	17520,166016	3,856740	43603,042969	0,028210
4	68880,000000	57890,308594	10989,691406	−0,450076	0,265054	24154,060547	8,198833	16635,289063	0,007804
5	517348,500000	560558,312500	−43209,812500	3,034193	−1,042154	36519,644531	19,982437	−192732,781250	0,394775
6	30264,000000	43534,156250	−13270,156250	−0,549586	−0,320056	14679,734375	2,420252	−15172,018555	0,002398
7	24308,000000	−11510,732422	35818,734375	−0,931133	0,863893	19665,970703	5,109980	46216,097656	0,039932
8	0,000000	6865,665527	−6865,665527	−0,803756	−0,165589	29473,783203	12,679496	−13879,150391	0,008089
9	302615,000000	255867,062500	46747,937500	0,922210	1,127488	13346,864258	1,833545	52152,117188	0,023421
10	455745,000000	416986,437500	38758,562500	2,039017	0,934797	20986,066406	5,952830	52108,113281	0,057806
11	36806,000000	50881,167969	−14075,167969	−0,498660	−0,339471	12295,106445	1,409970	−15432,205078	0,001740
12	380626,000000	329004,687500	51621,312500	1,429167	1,245027	18309,621094	4,300997	64126,687500	0,066640
13	0,000000	−1393,591675	1393,591675	−0,861005	0,033611	22036,017578	6,662284	1942,194824	0,000089
14	26767,500000	55095,511719	−28328,011719	−0,469448	−0,683228	13874,701172	2,059216	−31900,251953	0,009470
15	33121,500000	45034,687500	−11913,187500	−0,539185	−0,287328	10761,227539	0,854521	−12773,662109	0,000913
16	9837,500000	45711,902344	−35874,402344	−0,534491	−0,865235	19152,716797	4,797058	−45605,937500	0,036881
17	0,000000	49183,089844	−49183,089844	−0,510431	−1,186221	12053,177734	1,317454	−53723,175781	0,020269
18	3787,000000	31434,568359	−27647,568359	−0,633455	−0,666817	25984,638672	9,640365	−45530,246094	0,067660
19	137132,500000	181922,750000	−44790,250000	0,409661	−1,080272	18847,197266	4,614717	−56455,671875	0,054728
20	169589,000000	146748,609375	22840,390625	0,165850	0,550875	25761,769531	9,459234	37202,746094	0,044402
21	203844,500000	199061,875000	4782,625000	0,528462	0,115350	23490,330078	7,702166	7043,419434	0,001323
22	102502,000000	89345,914063	13156,085938	−0,232040	0,317305	12774,961914	1,598913	14536,040039	0,001667
23	171786,000000	117132,953125	54653,046875	−0,039433	1,318147	20211,140625	5,451424	71687,304688	0,101477
24	23838,000000	354,105560	23483,894531	−0,848891	0,566395	19622,796875	5,083339	30262,208984	0,017046
25	0,000000	88470,695313	−88470,695313	−0,238107	−2,133777	18716,539063	4,537632	−111112,648438	0,209064
26	134214,000000	82403,500000	51810,500000	−0,280162	1,249590	10352,226563	0,718894	55255,105469	0,015817
27	55858,000000	67571,953125	−11713,953125	−0,382967	−0,282523	17488,029297	3,839070	−14248,854492	0,003002
28	364299,000000	400780,093750	−36481,093750	1,926682	−0,879868	31462,785156	14,583098	−86005,632813	0,353955

Source: Authors’ calculations

In Table 8, the analysis proved the lack of autocorrelation of residuals and statistics value *d* = 1.947 and this means that there was enough data to calculate a statistically significant linear model in multiple regression procedure.

**Table 8:** The *d*Durbin-Watson model verification

Feature	*d* Durbin-Watson	Residual serial correlation
Estim.	1,947020	0,006487

Source: Authors’ calculations

**Fig. 5:**
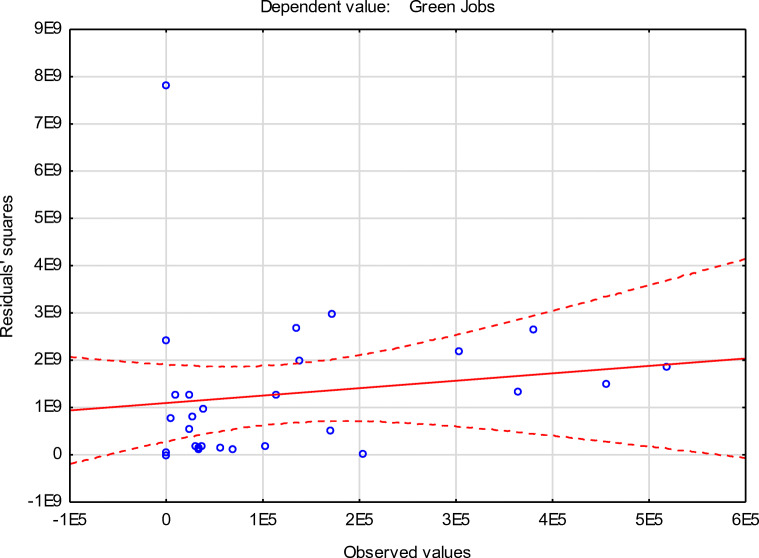
Observed values with residuals’ squares Note: *α* = 0.95 and *p*<0.000001 for the regression. Source: Authors’ calculations

Figure 5 proved the homoscedasticity assumption, which was fulfilled, supporting the model's linearity. The assumption is met because the points on the graph form an even cloud, without any characteristic pattern of points.

**Fig. 6:**
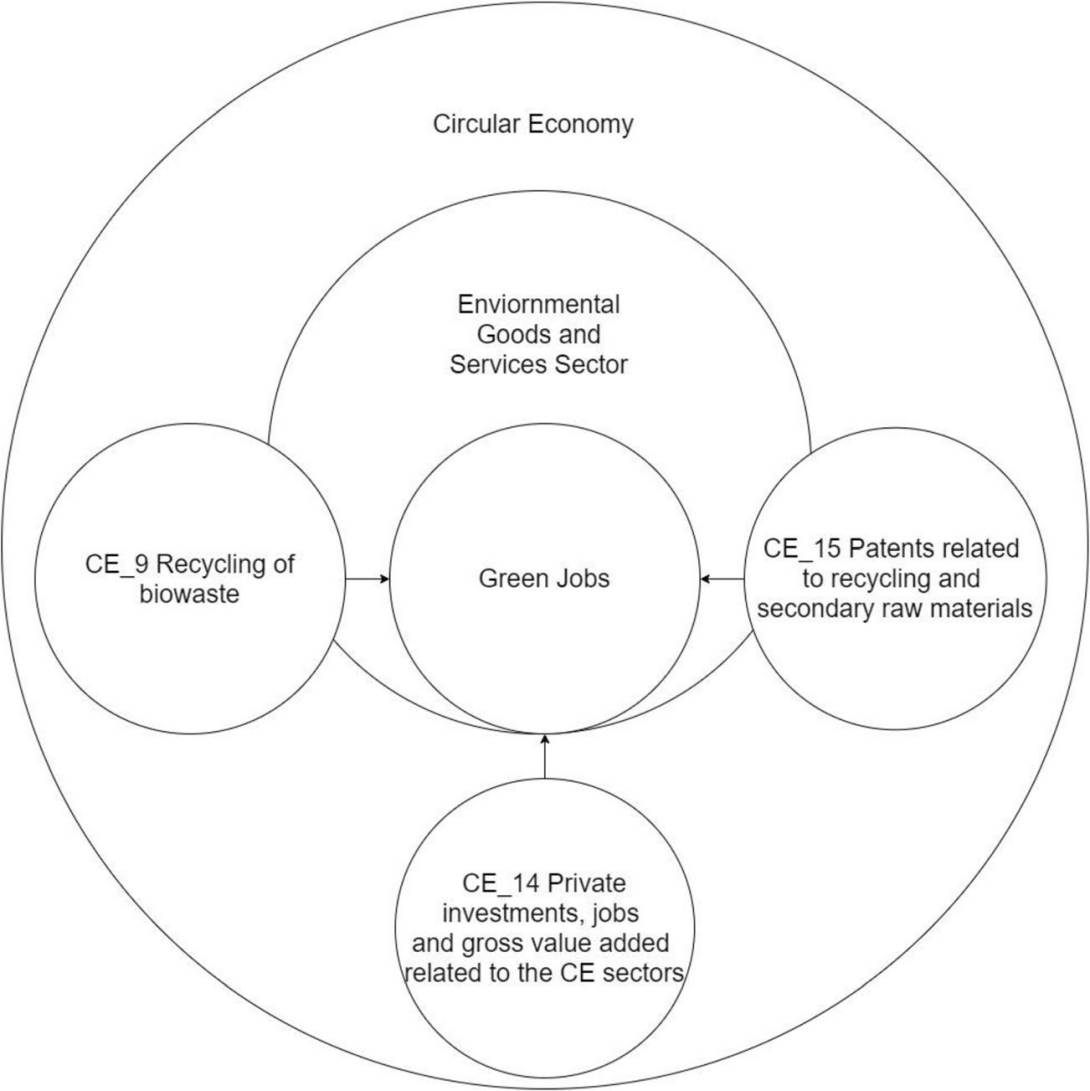
Green Jobs creation model for circular economy Source: Authors’ elaboration

The linear model (Eq. 1) presents the two sets of variables. This equation presents regression results, reducing the number of variables that were used in the final model. Regression allowed the description of statistically significance relations between GJs (*Employment in the Environmental Goods and Services Sector*) and CE_14; CE_15; CE_9. Variables’ dependencies create a model presented in Eq. 1 and Figure 6. The shape of the proposed model underlines the circularity feature of described economy.

The EGSS is a part of the circular economy and in this sector majority of the GJs are visible (Dordmond et al. 2021). However, the GJs can be created in the other sectors of the economy. Obtained results prove the unequivocal character of the three variables with described variable GJs. Thus, CE_14 (*Private investments, jobs, and gross value added related to circular economy sectors*) represents crucial expenditures related to the development of EGSS. Also significant for the model is variable CE_15 (*Patents related to recycling and secondary raw materials*), which also indicates that technological progress combined with investments can influence CE_9 (*Recycling of biowaste*). All these variables used in the model prove that GJs are part of the labor market influenced in the same matter by the inventions and investments, but they are very specific because their aim is reducing the anthropopressure (negative impact of human activity on the natural environment). In this perspective, the GJs are part of the EGSS.

## Discussion

The GJs creation process in the literature is described mostly qualitatively what includes sectors of the economy (Bruyère and Filiberto 2013; Conlon et al. 2019):

1. “Generation of energy from renewable sources (solar, wind, biofuels) including manufacturing, installation, and maintenance;
2. Improving energy efficiency including services like home energy audits, home renovation and retrofitting, and manufacture and provision of products like energy efficiency appliances;
3. Pollution reduction/removal recycling, greenhouse gas reduction;
4. Natural resources conservation including organic agriculture, sustainable forestry, and stormwater management;
5. Environmental compliance, education, and training including regulations and public awareness”.

This list of the areas or factors influencing the GJs creation process then is not used in a mathematical method to predict or calculate the number of the GJs but to prove transformation toward the CE (McMahon et al. 2021; Rojas Arboleda et al. 2021). The majority of the quantitative publications document the number of GJs (Ferrão et al. 2015) or are focused on the gap between demand and supply of GJs (Song et al. 2021). However, in the literature, there is a similarity to be presented in this paper, graphical approach describing the relations between GJs and CE (Horbach et al. 2015); these works are qualitative in their methods (Battaglia et al. 2018). Therefore, this paper brings novelty to science and expands GJs creation process quantitative horizon. Contrary to model presented in Eq. 1 and Figure 6, the other publication uses one chosen indicator or factor like the final energy consumption from renewables and income in rural areas (Aceleanu et al. 2018) or utilized agricultural area (Unay-Gailhard and Bojnec 2019). The model presented in this paper is supported by findings of Luca et al. (2019) which used multilevel logistic regression and confirmed their hypothesis that “the resources efficiency actions a company is taking, the more likely it is for employees to have a green job” (Luca et al. 2019, p. 69). Contrary to Luca et al. (2019), this paper covers 2009 and 2019 for all EU member states, and based on secondary data from Eurostat, the linear model of the Green Jobs creation process has been proposed. This research proposed the Green Jobs creation econometric model connected to the CE. This model describes relations between theoretical assumptions of CE and empirical implications in the emerging GJs using indicators constructed upon the Sustainable Development Goals (SDGs). Also, other papers examine the number of GJs in the CE (Mehmet 1995; Moreno-Mondéjar et al. 2021), but their methods are based on the strategies which we have considered as processes closing the loops in CE (Figure 1).

The literature review also underlines a lack of uniform methods for measuring and forecasting the effects of Green Jobs creation. This research contributes to science by identifying the green indicators of CE required in the GJs creation. In the article, we proposed new approaches to the studied issues, summarized in the form of tables and figures. The method used in this study comprises the variables identified in earlier studies and that have been validated by the decision-makers in 28 EU countries (Luca et al. 2019, p. 70). The presented model in Figure 6 is a novelty because it combines factors influencing GJs creation in EGSS, from a CE perspective. This model summarizes our research as it identifies three of the most important variables for Green Jobs creation. Based on this model, policy-makers should enhance the investments from *private sectors, gross value added, and jobs that are characterized as CE sectors* (CE_14), which is the most important variable in the proposed model. The importance for CE is technology development which is also crucial for the *number of patents focusing on the recycling and the use of secondary raw materials* (CE_15). The proposed model can be used to predict the number of GJs created in the CE with certain accuracy (Eq. 1). There is a consistency between assumption presented in Figure 1 where CE is created along with the new processes and Green Jobs to cover material cycles. The interdependencies between CE, GJs, and EGSS, also proved the importance of the SDGs. Similarly to the Luca et al. (2019), we introduced Green Jobs as an area between core elements of revised definitions and EGSS elements in Figure 3, what allowed us to use linear regression as main statistical methods in this study.

## Conclusions and implications

In the CE, people prevent environmental damage, control pollution, and take measures to protect the enterprise’s environment (Tang et al. 2018; Liu et al. 2020). Issues such as development in harmony between humanity and nature, the SD, wise science, and technology are gaining strategic importance. These concepts are crucial for CE development and indicate the need for agreement between the participants of economic processes at the various levels. Therefore, it is assumed that CE is related to the idea of “green civilization” coined by Norgaard (1994), where societies’ wisdom translates into civilization development regarding the natural environment and economic benefits for all (Vokoun and Jílková 2020; Wilts et al. 2021).

In the CE, the labor market can be shaped by activities at the macro and micro levels associated with GJs creation. A post-industrial civilization requires a different approach to employees. The honesty of the organization toward its stakeholders, following the proclaimed principles and values, and high moral standards, enforce enterprises toward the natural environment protection. The GJs must appear not only in politicians’ messages but mainly in business leaders and employees’ minds. The recommended approach is an integrated one toward GJs, where the organization’s standards translate into expectations toward employees and achieving specified SDGs. The implementation of the CE indicators and measurement of changes effects in culture and awareness at enterprises, employees, and individual recipient’s level allows to closing production cycles. The EGSS is a special environmental sector of the economy where specifically but not exclusively GJs are created. Besides that, this sector aims to reduce or eliminate environmental pressures. The GJs number is increasing due to technological changes and growing investments in EP and RM.

The main findings revealed in the proposed explanatory model of GJs creation are reflected in striving to implement the idea of CE. The model is indicating which strategic management fields can support a CE and enhance employment in EGSS which in this paper is described as GJs. Presented calculations are rare in the field of the CE scientific discussion about factors of GJs creation. The authors understand that each of the used indicators represents a process that supports the transformation toward the CE in the EU. The limitation of this study lies in the initial number of variables describing the CE proposed by Eurostat and adopted to this study. On the other hand, this set is comparable and widely accepted due to the Eurostat methodology. These features allow repeating the whole research procedure with scientific objectivity. Then authors could not choose arbitrary variables, other than those adopted in this study. Another limitation comes from the geographical characteristic of variables; they represent EU member states’ context only.

When pointing to the possibilities of implementing the CE model in practice, it should be considered that the GJs are next to the pillar of this concept, and they are necessary to achieve SDGs. Green Jobs act as a CE implementation tool and are a strategic goal for organizations in the economic model. The natural environment can be protected and restored by increasing the number of GJs. The GJs importance is not only based on protecting and developing the natural environment. Green Jobs are essential to employees because they ensure decent work and shape their destiny and the environmental protection level. Practitioners and entrepreneurs can focus also on the *recycling of biowaste* (CE_9) processes which also contribute to the GJs creation as presented in Figure 6. The simplicity of the proposed model can be suitable for the business because it translates theoretical findings into business language and is easy to implement business solutions. The GJs creation processes and factors are important because they offer a combined solution for climate, economic, and social crises.

The future research direction can be based on the replication of the research, the factor analysis usage; this type of method could better describe variability among observed and correlated variables used in this research. This study presents the dynamic changes in the years 2009–2019 for the GJs creation process in CE and satisfies the demand for similar longitudinal studies (Luca et al. 2019) which should be continued in the future (see Appendix Table 9).

## Data Availability

Not applicable

## Appendix

**Table 9:** Appendix. Regression statistics summary

Statistical feature	Values
*R* multiple	0,969348325
Multiple *R*^2^	0,939636174
Corrected *R*^2^	0,922389367
*F*(6,21)	54,4817458
*p*	0,0000000000101275195
Err. std. estimation	0.41462

Source: Authors’ calculations
